# Decision support tool for differential diagnosis of Acute Respiratory Distress Syndrome (ARDS) vs Cardiogenic Pulmonary Edema (CPE): a prospective validation and meta-analysis

**DOI:** 10.1186/s13054-014-0659-x

**Published:** 2014-11-29

**Authors:** Christopher N Schmickl, Sonal Pannu, Mazen O Al-Qadi, Anas Alsara, Rahul Kashyap, Rajanigandha Dhokarh, Vitaly Herasevich, Ognjen Gajic

**Affiliations:** Multidisciplinary Epidemiology and Translational Research in Intensive Care (METRIC), Division of Pulmonary and Critical Care Medicine, Mayo Clinic, 200 First Street SW, Rochester, MN 55905 USA; University Witten-Herdecke, Alfred-Herrhausen-Straße 50, 58448 Witten, Germany; Harvard School of Public Health, 677 Huntington Avenue, Boston, MA 02115 USA; Lahey Clinic, Pulmonary and Critical Care, 41 Burlington Mall Road, Burlington, MA 01805 USA

## Abstract

**Introduction:**

We recently presented a prediction score providing decision support with the often-challenging early differential diagnosis of acute lung injury (ALI) vs cardiogenic pulmonary edema (CPE). To facilitate clinical adoption, our objective was to prospectively validate its performance in an independent cohort.

**Methods:**

Over 9 months, adult patients consecutively admitted to any intensive care unit of a tertiary-care center developing acute pulmonary edema were identified in real-time using validated electronic surveillance. For eligible patients, predictors were abstracted from medical records within 48 hours of the alert. Post-hoc expert review blinded to the prediction score established gold standard diagnosis.

**Results:**

Of 1,516 patients identified by electronic surveillance, data were abstracted for 249 patients (93% within 48 hours of disease onset), of which expert review (kappa 0.93) classified 72 as ALI, 73 as CPE and excluded 104 as “other”. With an area under the curve (AUC) of 0.81 (95% confidence interval =0.73 to 0.88) the prediction score showed similar discrimination as in prior cohorts (development AUC = 0.81, *P* = 0.91; retrospective validation AUC = 0.80, *P* = 0.92). Hosmer-Lemeshow test was significant (*P* = 0.01), but across eight previously defined score ranges probabilities of ALI vs CPE were the same as in the development cohort (*P* = 0.60). Results were the same when comparing acute respiratory distress syndrome (ARDS, Berlin definition) vs CPE.

**Conclusion:**

The clinical prediction score reliably differentiates ARDS/ALI vs CPE. Pooled results provide precise estimates of the score’s performance which can be used to screen patient populations or to assess the probability of ALI/ARDS vs CPE in specific patients. The score may thus facilitate early inclusion into research studies and expedite prompt treatment.

**Electronic supplementary material:**

The online version of this article (doi:10.1186/s13054-014-0659-x) contains supplementary material, which is available to authorized users.

## Background

With an estimated 190,600 new cases each year resulting in 74,500 deaths and 3.6 million hospital days, acute respiratory distress syndrome (ARDS [1], formerly known as acute lung injury [ALI] [2]) poses a major health burden on US society [3]. In the early stages ARDS can be difficult to differentiate from cardiogenic pulmonary edema (CPE) [4,5], which may delay initiation of critical treatment measures (for example, lung-protective ventilation, prone positioning, neuromuscular blockade) [6-10], lead to unnecessary testing and preclude timely enrollment into research studies [11-13].

In both practice and research, the diagnosis of ARDS primarily rests on clinical judgment [4,14,15], which is limited by its subjectivity and substantial inter-rater variability. Therefore, we recently presented a prediction score providing objective decision support for early differential diagnosis of ARDS versus CPE based on routinely available clinical data (free download of calculator [16]) [17]. To facilitate clinical adoption of this prediction score our objective was to prospectively validate its performance in an independent cohort of consecutive patients.

## Materials and methods

During a 9-month period (March 2010 through January 2011) all adult patients admitted to any ICU at a tertiary care center in Rochester, MN, USA, were screened for new onset of acute pulmonary edema (defined as arterial partial pressure of oxygen (PaO_2_)/inspired oxygen fraction (FiO_2_) ratio <300 for arterial blood gas, and pulmonary edema or bilateral infiltrates on chest radiograph as read by radiologists, both within 24 h) using a previously validated electronic surveillance system [7]. After being alerted about these patients via email and the paging system in real time, research staff reviewed the electronic medical records (EMR) for potential eligibility within 48 h. Patients were excluded if they had been enrolled in the previously reported development cohort (DC) or retrospective validation cohort (RVC) [17], or if research staff could not review their medical records within 48 h of being alerted. The other exclusion criteria at this stage were: no research authorization, second or later episodes of acute pulmonary edema after enrollment, chest radiograph findings clearly inconsistent with ALI (for example, lower zone opacities only, chronic bilateral opacities; as emphasized in the Berlin definition of ARDS [1]), substantial missing data, mechanical ventilation prior to (>6 hours) development of acute pulmonary edema [17].

If patients were found potentially eligible, research staff (AA and CS) directly abstracted most of the data necessary for calculation of the prediction score from the EMR into a predesigned access® form (some data at low risk of differential measurement bias such as age were retrieved retrospectively from a validated ICU database; details in Additional file 1) [7].

Post-hoc expert review served as the gold standard: after patients’ discharge or death a board-certified critical care physician (SP) and a clinical critical care fellow (MA) - blinded to each other as well as to the details and results of the prediction score - reviewed all included patients after discharge or death, taking into account all available information in the EMR including the course of illness and response to therapeutic intervention using the following definitions (with disagreements resolved via super-review by an experienced investigator [RK]) [17]: ALI was defined according to the American-European Consensus Conference (AECC) statement as acute (<24 h) hypoxemia (PaO_2_/FiO_2_ ratio <300) with bilateral lung infiltrates consistent with pulmonary edema on frontal chest radiograph without any evidence of left atrial hypertension [2]. Left atrial hypertension was excluded by echocardiographic findings (E/E’ <15), brain natriuretic peptide (BNP) levels (BNP <250 pg/mL in the absence of renal failure), and venous filling pressures (pulmonary wedge pressure ≤18 or central venous pressure ≤12 mmHg in the absence of pulmonary hypertension).

CPE was defined by a combination of clinical signs (jugular venous distension, systolic hypertension); radiographic (cardiothoracic ratio >0.53 and vascular pedicle width >0.65 mm), electrocardiogram (new ST-segment and T-wave changes), laboratory (elevated troponin T level >0.1 ng/mL), and hemodynamic findings (pulmonary wedge pressure >18 mmHg, central venous pressure >12 mmHg, decreased ejection fraction <45%, E/E’ >15, presence of severe left-sided valvular heart disease (aortic or mitral stenosis or regurgitation)); and a brisk response (hours) to appropriate therapy (preload/afterload reduction, treatment of ischemia or inotropic agents). Patients meeting both criteria were classified as ALI + CPE; those meeting neither definition where classified as other and excluded from the main analysis.

During the study the Berlin definition for acute respiratory distress syndrome (ARDS) [1] was published replacing the AECC definition for ALI [2]. We tried to account for this by performing sensitivity analysis, in which ALI patients with a positive end-expiratory pressure (PEEP) measurement ≥5 cmH_2_O within 12 h of acute pulmonary edema were classified as having ARDS (Berlin definition) [1].

The Mayo Clinic Institutional Review Board (IRB) approved the use of medical records for research and waived the requirement for written informed consent as doing so would not have been feasible, and the risk from this study for patients was minimal (IRB number 10-000348). Only patients who gave permission to use their medical record for research (research authorization) were included in this study.

### Statistical analysis

Agreement between post-hoc expert reviewers was evaluated by the kappa statistic. Patients with both ALI and CPE were treated as ALI cases in all analyses unless stated otherwise. Data were summarized as median (IQR) or percent (number) for each group (ALI versus CPE). Univariable analyses were carried out using the Wilcoxon rank-sum test and the chi-squared or Fisher exact test as appropriate. For multivariable analyses a single imputation method was used (this value was assumed for variables including 0 in the normal reference range, otherwise, the overall median was chosen) [17].

Discrimination of the prediction score as judged by the area under the receiver operating characteristic curve (AUC) was formally compared with the results from the two previous cohorts [17] (prospective validation cohort (PVC) versus development cohort (DC) and retrospective validation cohort (RVC)) using the chi-squared test. A priori we decided to assess calibration using: 1) the Hosmer-Lemeshow (HL) test, and 2) the chi-squared test for significant deviations of observed versus expected numbers of ALI cases across eight score categories, which had been previously defined during score development [17] with:

$$ {\mathrm{N}}_{\mathrm{ALI}\hbox{-} \mathrm{expected},\mathrm{P}\mathrm{V}\mathrm{C},\mathrm{i}} = {\mathrm{N}}_{\mathrm{total},\mathrm{P}\mathrm{V}\mathrm{C},\mathrm{i}} \times {\mathrm{Probability}}_{\mathrm{ALI},\mathrm{D}\mathrm{C},\mathrm{i}} $$

for each score category i.

In sensitivity analyses the AUC was estimated: 1) excluding patients with both conditions (ALI + CPE), 2) comparing ARDS (Berlin definition) [1] against CPE, 3) using bootstrap analysis with 10,000 samples to assess the robustness of results, and 4) comparing ALI versus CPE + other and CPE versus ALI + other patients.

In post-hoc analysis we tried to identify predictors of other versus ALI or CPE. We further compared the observed versus expected number of ALI cases across the eight pre-defined score categories in the (previously published) retrospective validation cohort [17], and driven by the results assessed in logistic regression models if referral patient status (as a proxy for ALI versus CPE prevalence) is an independent predictor of ALI versus CPE. Lastly, post-hoc meta-analysis was performed in which individual patient data from the current and previous cohorts were combined to obtain pooled estimates for the AUC (DC + RVC + PVC), the sensitivity/specificity at different cutoff points (DC + RVC + PVC), and the probability of ALI versus CPE for each of the eight pre-defined score ranges (DC + PVC). Analyses were performed in JMP 9.0.3 and SAS 9.3 (SAS Institute Inc., Cary, NC, USA) using two-sided *P*-values <0.05 to judge statistical significance.

## Results and discussion

**Figure 1:**
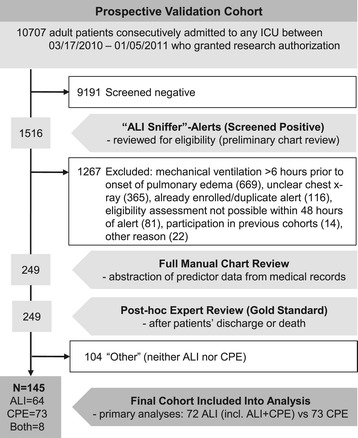
**Study flowchart.** ALI, acute lung injury; CPE, cardiogenic pulmonary edema.

Of 1,516 patients identified by electronic surveillance, data were abstracted from the EMR for 249 patients (Figure 1). Agreement between post-hoc expert reviewers was excellent (kappa = 0.93). A total of 104 patients (42%) were classified as “other”, as they did not meet the criteria for ALI or CPE (general characteristics in Additional file 2: Table S1), thus the final prospective validation cohort (PVC) consisted of 145 patients (64 ALI, 8 ALI + CPE, 73 CPE). For 50% of these patients predictor data were abstracted from the EMR within 12 h of onset of acute pulmonary edema (93% within 48 h). Patients with ALI were significantly younger, more likely to require invasive mechanical ventilation and had higher hospital mortality than CPE patients, with 81% meeting ARDS criteria based on the Berlin definition within 12 h of disease onset (Table 1).

**Table 1:** **General characteristics**

		**Prospective validation cohort**				
	**n**	**ALI/ALI + CPE (n = 72)**		**CPE (n = 73)**		***P*** **-value**
**General Characteristics**						
Age, years		61	(49 to 69)	73	(63 to 79)	<0.001
<45 years		13	(9)	7	(5)	0.25
Female sex		50	(36)	40	(29)	0.21
Body mass index		27	(23 to 35)	27	(24 to 32)	0.73
APACHE III score		53	(38 to 73)	52	(36 to 69)	0.70
Charlson score		2	(0 to 3)	2	(1 to 5)	0.07
Smoking	132	37	(25)	46	(30)	0.30
Referral patient		82	(59)	75	(55)	0.33
Admission ICU						0.04
medical		61	(44)	78	(57)	
surgical		10	(7)	10	(7)	
mixed		29	(21)	12	(9)	
Non-invasive ventilation only		4	(3)	23	(17)	0.001
Invasive mechanical ventilation		92	(66)	67	(49)	<0.001
ARDS (Berlin definition [1])*		81	(58)	na		na
Hospital mortality		47	(34)	15	(11)	<0.001
**ALI risk factors**						
Sepsis		57	(41)	23	(17)	<0.001
Pancreatitis		0	(0)	1	(1)	0.32
Pneumonia		33	(24)	11	(8)	0.001
Aspiration		8	(6)	0	(0)	0.01
**CPE risk factors**						
History of coronary artery disease		11	(8)	47	(34)	<0.001
History of congestive heart failure		11	(8)	33	(24)	0.002
New ST-changes/left-bundle branch block	110	8	(4)	31	(19)	0.003
**Other predictors**						
Alcohol abuse	135	19	(13)	6	(4)	0.02
Chemotherapy	144	21	(15)	6	(4)	0.006
SpO_2_/FiO_2_ ratio at 6 h after onset of acute pulmonary edema	141	181	(129 to 267)	240	(175 to 436)	0.002
<235	141	71	(48)	43	(31)	0.001

Data are presented as median (IQR) or percent (number); Total number was 145 unless noted otherwise. *ALI patients with positive end-exiratory pressure ≥5cmH_2_O within 12 h of acute pulmonary edema (94% met this criterion within 48 h). ALI, acute lung injury; ARDS, acute respiratory distress syndrome; CPE, cardiogenic pulmonary edema; APACHE, acute physiology and chronic health evaluation; FiO_2_: inspired oxygen fraction; SpO_2_, peripheral oxygen saturation; na, not applicable.

**Figure 2:**
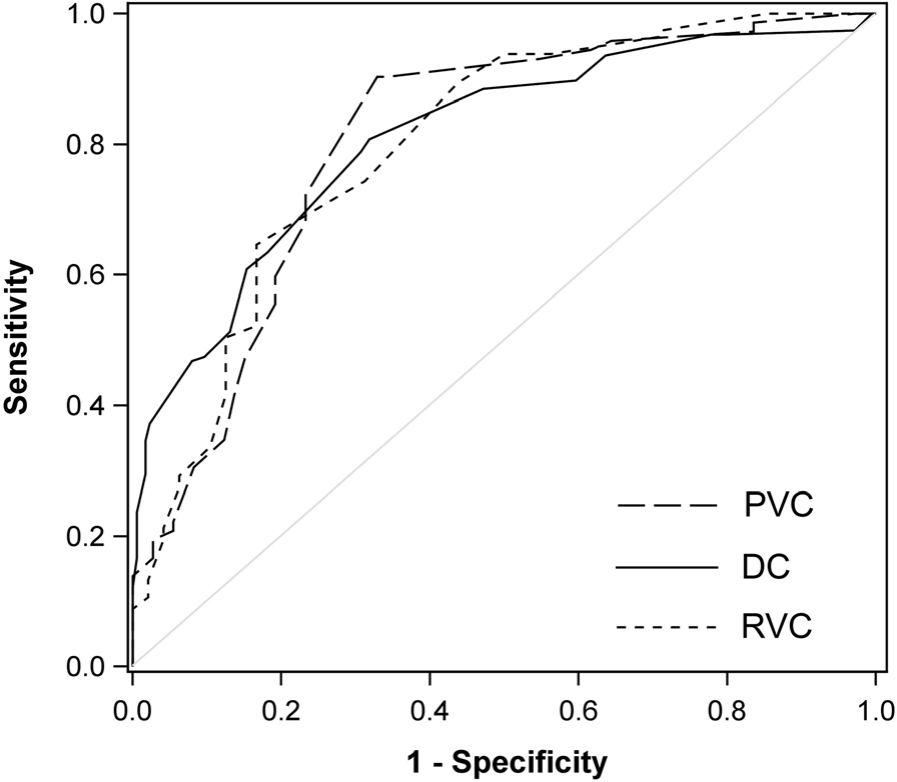
**Overlying receiver operating characteristic curves.** Development cohort (DC; AUC = 0.81, 95 CI = 0.77 to 0.86), retrospective validation cohort (RVC; AUC = 0.80, 95% CI = 0.72 to 0.88) and prospective validation cohort (PVC; AUC = 0.81, 95% CI = 0.73 to 0.88).

Based on the AUC, the prediction score discriminated equally well between ALI and CPE as in the two previous cohorts (AUC_PVC_ = 0.81, 95% CI = 0.73 to 0.88 compared to AUC_DC_ = 0.81, P = 0.85 and AUC_RVC_ = 0.80, *P* = 0.97; Figure 2). In sensitivity analyses the AUC did not change when (1) excluding patients with both ALI + CPE (AUC = 0.82, 95% CI = 0.75 to 0.89; n = 137), (2) predicting ARDS (Berlin definition) [1] versus CPE (AUC = 0.80, 95% CI = 0.73 to 0.88; n = 131), or (3) bootstrapping the analysis (AUC = 0.81, 95% CI =73 to 0.88), but was substantially lower when comparing ALI versus CPE + Other (AUC = 0.73, 95% CI = 0.66 to 0.79; sensitivity/specificity in Additional file 3: Table S2) or CPE versus ALI + Other (AUC = 0.71, 95% CI = 0.64 to 0.78; sensitivity/specificity in Additional file 3: Table S2). In post-hoc analysis the only significant predictor of the category, other, compared to ALI or CPE patients was the SpO_2_/FiO_2_-ratio at six h after onset of respiratory failure (odds ratio (OR) = 0.37, 95% CI = 0.22 to 0.64; *P* <0.001; see Additional file 4: Table S3).

**Figure 3:**
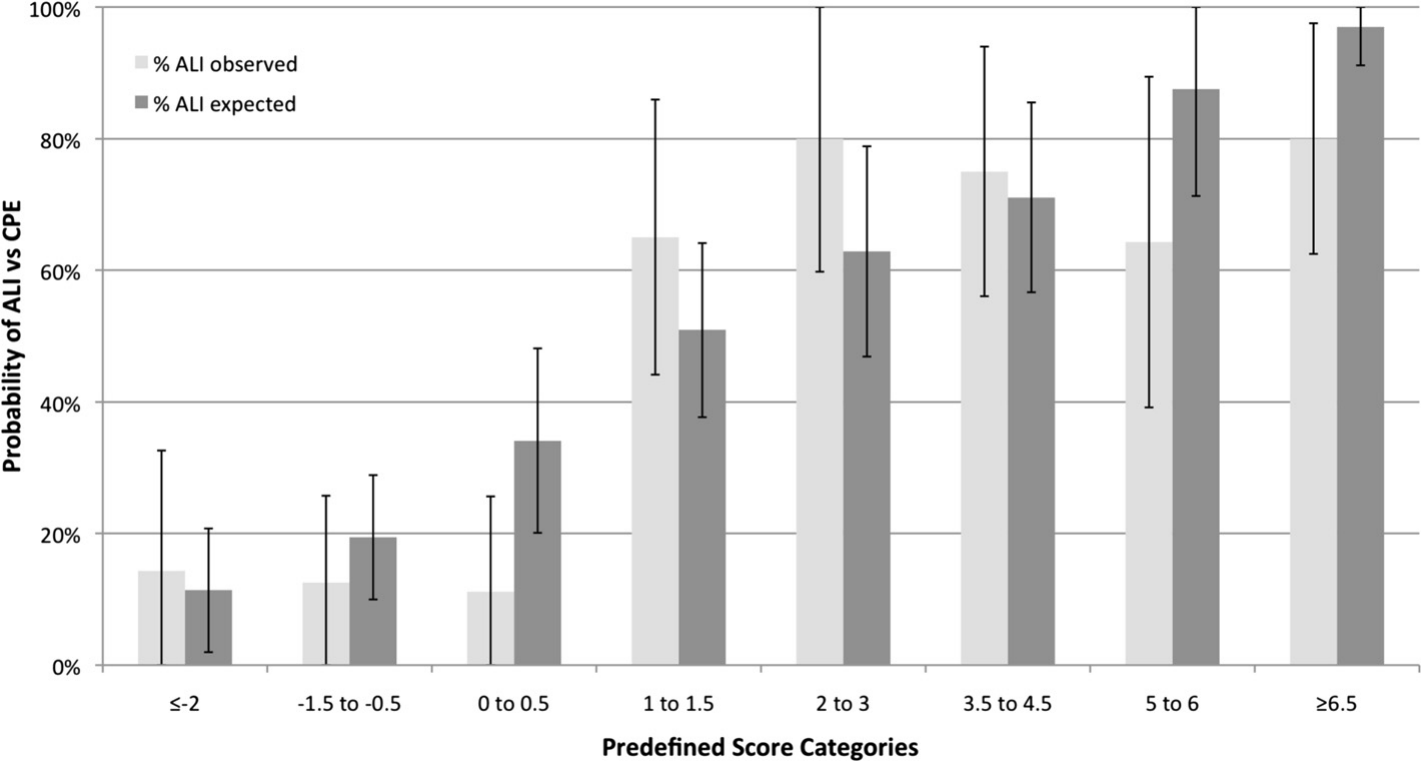
**Percent (and 95% CI) acute lung injury (ALI) cases observed (light gray bars) versus expected (dark gray bars) for each of the eight previously published prediction score ranges.** The number/percent of expected ALI cases is based on the observed percentages of ALI cases across the eight score categories in the development cohort, which is used by the online calculator to translate a given patient’s score sum into the predicted probability of ALI versus cardiogenic pulmonary edema (CPE) [17]. Based on the Chi-squared test with 7 degrees of freedom there were no significant differences in observed versus expected number of ALI cases overall (*P* = 0.49).

Based on the HL test the score was not well-calibrated when modeled as a linear or quadratic function of the log-odds of ALI (*P* = 0.01 and *P* = 0.047), but clinically the differences between observed and expected cases across deciles of predicted probabilities were small (Additional file 5). Also, when compared across the eight pre-defined score ranges the number of observed versus expected ALI cases did not differ in the prospective validation cohort (Figure 3; *P* = 0.49).

**Figure 4:**
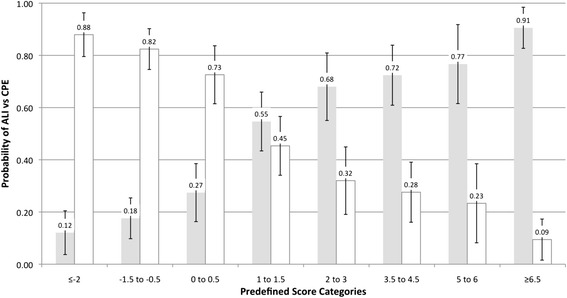
**Pooled estimates for the probability (and 95% CI) of acute lung injury (ALI) versus cardiogenic pulmonary edema (CPE) (gray bars) and CPE versus ALI (white bars) across eight previously published prediction score ranges (based on combined data from the development and prospective validation cohort (n = 477).** Overall prevalence of ALI versus CPE = 48%.

In post-hoc analysis, we found significantly more ALI cases than expected across the eight score ranges in the retrospective validation cohort (*P* = 0.004). We hypothesized that those results may be explained by higher prevalence of ALI versus CPE in referral patients, but referral status was not an independent predictor of ALI versus CPE in the prospective validation cohort (*P* = 0.14; details in Additional file 6). Based on pooled data the AUC was 0.81 (95% CI = 0.78 to 0.84). Pooled estimates for (1) sensitivity/specificity at various cutoffs are shown in Additional file 7: Table S4, and (2) the probabilities of ALI versus CPE (and vice versa) across the eight score ranges are presented in Figure 4.

This prospective validation provides further evidence that the prediction score differentiates well between ARDS and CPE patients. The score can be used in two different ways: (1) to screen a patient population (for example, for early enrollment of ARDS patients into a clinical trial) by using a cutoff value: given the similar settings and results we decided to pool the data from all three currently existing cohorts to obtain more precise estimates for the sensitivity/specificity at different cutoffs (Additional file 7: Table S4); (2) to estimate the probability of ARDS versus CPE for a specific patient based on the patient’s score result. This probability could be derived from a logistic regression function regressing the observed outcomes against the prediction score, but based on the goodness-of-fit testing it may be better to avoid this approach (details in Additional file 5). Thus, instead we suggest estimation of this probability as the observed proportion of patients with ARDS in the corresponding score range. However, either approach essentially provides a positive predictive value, which depends on the prevalence of ARDS versus CPE (P_ARDS prior_) in the underlying cohort. Thus, the estimated proportions of ARDS across the eight score ranges shown in Figure 4 are only based on the combined data from the development and prospective validation cohort (P_ARDS prior_ approximately = 0.5 in both cohorts), and should only be applied to patients from populations with similar prevalence of ARDS versus CPE. Estimates from the retrospective validation cohort could be used to estimate the probability of ARDS versus CPE in patients from populations with a P_ARDS prior_ approximately = 0.7 (see Additional file 6).

Other tests for differentiating ARDS versus CPE have been proposed. For example low levels of BNP are indicative of ARDS as opposed to CPE [18,19], but the high prevalence of renal failure among critically ill patients limits its usefulness [18,20]. The value of other more investigational laboratory markers such as Clara Cell protein 16 or Copeptin is less clear, and they likely suffer from the same limitations as BNP [21,22].

Ultrasonography is used increasingly commonly in clinical practice [23]. Its advantages are that it can be performed quickly, has virtually no side effects, and may provide guidance even in patients for whom no past medical history is available (which would preclude calculation of the clinical prediction score). However, sonographic visibility is often limited in the ICU due to the presence of multiple devices, chest trauma, surgical incisions or obesity. Furthermore its diagnostic value is operator-skill dependent, and its application requires substantial pre-training, the availability of expensive equipment and direct patient contact (prohibiting its use in e-ICU (electronic intensive care unit) settings).

Additional tests that have been proposed to differentiate ARDS versus CPE include the pulmonary vascular permeability index [24] and the fluid-to-plasma protein ratio [25], but these methods require the presence of an invasive thermodilution device or direct sampling of lung water, respectively, restricting their practical usefulness to selected patients only.

Strengths of our study include the enrollment of consecutive patients using a prospective study design: 95% of patients identified by electronic surveillance were evaluated for eligibility and predictor data were collected early after the onset of acute pulmonary edema, thus, minimizing the risk of selection and measurement bias, respectively. A further strength is that several sensitivity analyses demonstrated robustness of results thus providing further evidence for internal validity.

The main weakness is that generalizability of our results may be limited because this and the previous (development + retrospective validation) cohorts were recruited at the same institution. Furthermore, use of the score to predict an individual’s risk of ARDS versus CPE is restricted to settings with a similar underlying prevalence as discussed above. Another limitation is that during the course of this study the Berlin definition for ARDS [1] was published replacing the previous ALI definition [2]. The biggest difference is the newly introduced requirement of a PEEP of ≥5 cmH_2_O [1]. While the time frame has not been defined, as expected, the majority of included ALI patients met this criterion within 12 h of onset of acute respiratory failure (94% within 48 h), and while all main analyses compared ALI versus CPE patients, the results did not change when limiting ALI patients to this subset, suggesting that the results apply equally to ARDS versus CPE patients.

Further, sample size of the validation cohort was somewhat limited, but results did not change when using a bootstrap procedure (simulating the sampling of 10,000 cohorts of the same size from the underlying population) making it unlikely that random sampling variability had a substantial effect on the results.

While we believe that clinically it is usually relatively easy to distinguish patients with pulmonary edema (ALI or CPE) from those with other etiologies mimicking acute pulmonary edema at the onset illness (for example, atelectases, acute chronic interstitial lung disease, diffuse alveolar hemorrhage), in this study we deferred this evaluation to the post-hoc review by design for two reasons: 1) to minimize the potential for differential measurement bias we tried to separate collection of predictor data and outcome assessment as much as possible, and thus, did not want the person who collected the predictor data to evaluate the potential cause of the patients’ condition; 2) due to the lack of expert clinical knowledge by the research personnel who abstracted the predictor data we intentionally decided to be rather over-inclusive at the stage of data abstraction to prevent possible exclusion of ALI/CPE cases (which would have reduced internal validity). This approach limits our results in that the main analysis assumes that all patients with etiologies other than ALI/CPE can be recognized at initial presentation. The more this assumption fails the more we overestimated the score’s true performance. While the study design did not allow us to test this assumption, the sensitivity analyses comparing ALI versus CPE + other (and CPE versus ALI + other) indicates that in the worst-case scenario (if it was impossible initially to sort out any patients classified as other) the prediction score would still perform moderately well (Additional file 3: Table S2 shows the increased trade-offs for a high sensitivity or specificity as compared to the best-case scenario in Additional file 7: Table S4).

Another limitation is that ARDS and CPE may coexist [1,26]. Because our focus was to identify patients with any ARDS component we decided to include ALI + CPE patients in the ALI group for all main analyses, thereby also concurring with previous practice [17,26]. The number of patients with both conditions was small (n = 8), thus, the results did not change when excluding these patients in a sensitivity analysis and as expected most of these patients had intermediate scores (data not shown). In addition our study is limited by the lack of a true gold standard for both ARDS and CPE and the fact that oxygenation in some patients is impaired simply by fluid overload without overt heart failure.

One promising future application of the prediction score would be its integration into EMR infrastructure to automate calculation. The results could be used in various ways: for example to provide near real-time decision support to (junior) physicians, to alert research staff, or for quality assurance (for example, sending pager messages to healthcare personnel if a patient at high risk of ARDS does not receive low tidal volume ventilation) [27]. However, for EMR integration one challenge will be to filter out patients who have neither ARDS nor CPE automatically, as the score’s performance will otherwise be reduced (see Discussion above). Future research should: 1) validate the score in a different institutional setting (preferentially using a multicenter design), 2) identify predictors of ARDS/CPE versus other etiologies, 3) assess the feasibility of integrating the prediction score into EMR infrastructure, 4) evaluate the value of combining the clinical prediction score with findings from ultrasound examinations (especially in patients with intermediate score results), and 5) assess the impact of early differential diagnosis on clinical patient outcomes.

## Conclusions

In this prospective validation the clinical prediction score again demonstrated good differentiation between ARDS and CPE. Pooled results provide precise estimates of the score’s performance which can be used to screen patient populations or to assess the probability of ARDS versus CPE in specific patients (if the underlying patient populations are similar). The clinical prediction score may thus facilitate early inclusion into research studies and expedite the initiation of critical treatment measures.

## Key messages

- The clinical prediction score differentiates ARDS versus CPE reliably well across different patients cohorts
- In populations with similar ARDS versus CPE prevalence, the percentage of ARDS/CPE patients across eight score ranges is similar and can be used to predict an individual patient’s risk of ARDS versus CPE (and vice versa)

## Additional files

Additional file 1
**Variable definitions.**
Additional file 2: Table S1. Characteristics of “other” patients. Additional file 3: Table S2. Sensitivity and specificity when including “other”. Additional file 4: Table S3. Post hoc analysis of predictors for other versus acute lung injury/cardiogenic pulmonary edema (ALI/CPE). Additional file 5
**Details about the Hosmer-Lemeshow test.**
Additional file 6
**Post hoc analysis of referral status.**
Additional file 7: Table S4. Sensitivity and specificity based on meta-analysis.

## Abbreviations

AECC: American European consensus conference
ALI: acute lung injury
ARDS: acute respiratory distress syndrome
AUC: area under the curve
BNP: brain natriuretic peptide
CPE: cardiogenic pulmonary edema
DC: development cohort
EMR: electronic medical records
FiO_2_: inspired oxygen fraction
HL: Hosmer-Lemeshow test
IRB: Institutional Review Board
PaO_2_: arterial partial pressure of oxygen
PEEP: positive end-expiratory pressure
PVC: prospective validation cohort
RVC: retrospective validation cohort
