# Supplementary material for: Decision support tool for differential diagnosis of Acute Respiratory Distress Syndrome (ARDS) vs Cardiogenic Pulmonary Edema (CPE): a prospective validation and meta-analysis
Source: Crit Care. 2014 Nov 29;18(6):659. doi: 10.1186/s13054-014-0659-x (PMC4277656; doi:10.1186/s13054-014-0659-x)
Supplement: Additional file 1 — Variable definitions. [file 13054_2014_659_MOESM1_ESM.docx]

**Additional file 1. Definitions of variables used for the prediction score**

| **Predictor Variable** | **Definition** | **Coding** |
| --- | --- | --- |
| **General Information** | | |
| **Age**  **(**at T0)**^1^** | Calculated as time from date of birth till onset of acute pulmonary edema | 1 = <45 years  0 = else |
| **Risk Factors for Cardiogenic Pulmonary Edema** | | |
| **History**  (>3 days before T0) of **Heart Failure^2^** | Diagnosed or suggested in EMR, or | 0 = no  1 = yes |
|  | Suggested by Echo findings (EF<45% or diastolic relaxation abnormalities of at least grade II) |  |
| **History**  (>3 days before T0) **of Coronary Artery Disease^2^** | Diagnosed (as CAD or ischemic Cardiomyopathy) in EMR, or | 0 = no  1 = yes |
|  | Suggested by heart catheterization report, or |  |
|  | Previous ischemic event like myocardial infarction, known Angina, etc |  |
| **ST Changes**  (within 12h before till 6h after T0)**^2^** | New ST Segment deviation of >/ 1mm in two consecutive leads, or | 0 = no / NA  1 = yes |
|  | New Left Bundle Branch Block  **Not:** “secondary ST changes” (e.g. RBBB) or “unspecific ST abnormalities” |  |
| **Risk Factors for Acute Lung Injury** | | |
| **Sepsis**  (within 24 h before T0)**^2^** | I. Suspected or documented infection  +  II. Systemic Inflammatory Response Syndrome SIRS (=at least two out of the following four are prevalent)   1. Temperature >38° or <36° C 2. Heart rate (HR) > 90/min 3. >20 respirations/min or PaCO2<32mmHg 4. Leucocytes <4,000/mm³ or >12,000/mm³ | 0 = no  1 = yes |
| **Pancreatitis**  (within 24 hours before T0)**^2^** | Two or more out of the following three:   1. Abdominal pain characteristic of acute pancreatitis 2. Serum amylase and/or lipase >/ 3 times the upper limit of normal 3. Characteristic findings of acute pancreatitis on CT | 0 = no  1 = yes |
| **Pneumonia**  (within the 5 days prior to T0)**^2^** | I. New or progressive radiographic infiltrate  +  II. High clinical suspicion of pneumonia: a) or b)  a) New cough, sputum, fever or WBC>12,000/mm³  b) Suggested or diagnosed in EMR  (not only DD, should be at least treated), or | 0 = no  1 = yes |
|  | I. NEW Abnormal chest radiograph of uncertain cause  +  II, Microbiological or serological evidence of definite  or probable pneumonia (result available till 6h after  SnifferTime!)  +  III. Low or moderate clinical suspicion of pneumonia |  |
| **Aspiration**  (within 48 hours before T0)**^2^** | Witnessed or suggestive history of gastric aspiration | 0 = no  1 = yes |
| **ALI Risk modifier and Miscellaneous** | | |
| **Alcohol Abuse^2^** | >2drinks per day (any alcoholic beverage), or | 0 = no  1 = yes |
|  | if previous alcoholic, sober for <1 year |  |
| **Chemotherapy^2^** | Currently taken or within the six months before T0 as of documentation in EMR | 0 = no  1 = yes |
| **SpO_2_ at 6h (/5h)^1^** | The value within 30 minutes before till 30 minutes after onset of acute pulmonary edema ( [T0+6h-30min;T0+6h+30min[ ) closest to this exact time  If NA, use T0 plus 5h as alternative time point | value |
| **FiO_2_ at “SpO_2_ at 6h (/5h)”^1^** | Closest value before “SpO_2_ at 6h (/5h)”, if not available before assume room air (FiO_2_ = 0.21) | value |

^1^ data at low risk of differential misclassification bias were electronically queried from *datamart* (intensive care database permanently saving information available in electronic medical records [Herasevich V et al. (2010). "Informatics infrastructure for syndrome surveillance, decision support, reporting, and modeling of critical illness." Mayo Clin Proc 85(3): 247-254.]) blinded to patients’ prediction score and final diagnosis retrospectively;

^2^ data abstracted manually from electronic medical records as soon as possible after alert from electronic screening (blinded to results of the prediction score and final diagnosis)

Abbreviations: T0 = time of manually asserted onset of acute pulmonary edema, defined as the time when both the results of an arterial blood gas with PaO_2_/FiO_2_ <300 AND a chest x-ray report consistent with pulmonary edema were first available; EMR = electronic medical record.
