# Supplementary material for: Decision support tool for differential diagnosis of Acute Respiratory Distress Syndrome (ARDS) vs Cardiogenic Pulmonary Edema (CPE): a prospective validation and meta-analysis
Source: Crit Care. 2014 Nov 29;18(6):659. doi: 10.1186/s13054-014-0659-x (PMC4277656; doi:10.1186/s13054-014-0659-x)
Supplement: Additional file 2: Table S1. — Characteristics of “other” patients. [file 13054_2014_659_MOESM2_ESM.docx]

**Additional file 2: Table S1. General characteristics of patients with a diagnosis “other” than ALI, CPE or ALI+CPE.**

|  | **Other (N=104)** | |  |
| --- | --- | --- | --- |
| **General Characteristics** |  |  |  |
| Age (years), n=102 | 69 | (57 to 77) |  |
| <45 years, n=102 | 9 | (9) |  |
| **ALI Risk Factors** |  |  |  |
| Sepsis | 40 | (42) |  |
| Pancreatitis | 0 | (0) |  |
| Pneumonia | 25 | (26) |  |
| Aspiration | 8 | (8) |  |
| **CPE Risk Factors** |  |  |  |
| History of coronary artery disease | 24 | (25) |  |
| History of congestive heart failure | 24 | (25) |  |
| New ST-changes/ left bundle branch block | 12 | (12) |  |
| **Other Predictors** |  |  |  |
| Alcohol Abuse | 8 | (8) |  |
| Chemotherapy | 15 | (16) |  |
| SpO_2_/FiO_2_-ratio at 6 hours after onset of acute pulmonary edema, n=100 | 250 | (187 to 452) |  |
| <235, n=100 | 36 | (36) |  |

Data are presented as median (IQR) or percent (number); Total N was 104 unless noted otherwise
