# Supplementary material for: Decision support tool for differential diagnosis of Acute Respiratory Distress Syndrome (ARDS) vs Cardiogenic Pulmonary Edema (CPE): a prospective validation and meta-analysis
Source: Crit Care. 2014 Nov 29;18(6):659. doi: 10.1186/s13054-014-0659-x (PMC4277656; doi:10.1186/s13054-014-0659-x)
Supplement: Additional file 3: Table S2. — Sensitivity and specificity when including “other”. [file 13054_2014_659_MOESM3_ESM.docx]

**Additional file 3: Table S2. Sensitivity and specificity at different cut-offs in the prospective validation cohort when including patients with “other” diagnosis than ALI, CPE.**

|  |  | **Prospective Validation Cohort**  **ALI vs CPE+other** | | | | |  |
| --- | --- | --- | --- | --- | --- | --- | --- |
| **Cut-off** |  | **Sensitivity**  **(95%-CI)** | |  | **Specificity**  **(95%-CI)** | |  |
| **> -1.5** |  | 96 | (88 to 99) |  | 25 | (18 to 32) |  |
| **> -0.5** |  | 93 | (84 to 97) |  | 35 | (28 to 43) |  |
| **> 0** |  | 90 | (80 to 96) |  | 51 | (43 to 58) |  |
| **> 1** |  | 72 | (60 to 82) |  | 62 | (54 to 69) |  |
| **> 2** |  | 68 | (56 to 78) |  | 64 | (56 to 71) |  |
| **> 3** |  | 56 | (43 to 67) |  | 72 | (65 to 79) |  |
| **> 4** |  | 42 | (30 to 54) |  | 81 | (75 to 87) |  |
| **> 5** |  | 31 | (21 to 43) |  | 88 | (82 to 92) |  |

“ALI” if > cut-off

|  |  | **Prospective Validation Cohort**  **CPE vs ALI+other** | | | | |  |
| --- | --- | --- | --- | --- | --- | --- | --- |
| **Cut-off** |  | **Sensitivity**  **(95%-CI)** | |  | **Specificity**  **(95%-CI)** | |  |
| **≤ -1.5** |  | 36 | (25 to 48) |  | 88 | (82 to 92) |  |
| **≤ -0.5** |  | 45 | (34 to 57) |  | 81 | (74 to 86) |  |
| **≤ 0** |  | 66 | (54 to 76) |  | 72 | (65 to 79) |  |
| **≤ 1** |  | 77 | (65 to 86) |  | 59 | (51 to 66) |  |
| **≤ 2** |  | 77 | (65 to 86) |  | 55 | (47 to 62) |  |
| **≤ 3** |  | 81 | (70 to 89) |  | 43 | (35 to 50) |  |
| **≤ 4** |  | 86 | (76 to 93) |  | 30 | (24 to 38) |  |
| **≤ 5** |  | 92 | (82 to 97) |  | 21 | (15 to 28) |  |

“CPE” if ≤ cut-off
