# Supplementary material for: Decision support tool for differential diagnosis of Acute Respiratory Distress Syndrome (ARDS) vs Cardiogenic Pulmonary Edema (CPE): a prospective validation and meta-analysis
Source: Crit Care. 2014 Nov 29;18(6):659. doi: 10.1186/s13054-014-0659-x (PMC4277656; doi:10.1186/s13054-014-0659-x)
Supplement: Additional file 4: Table S3. — Post hoc analysis of predictors for other versus acute lung injury/cardiogenic pulmonary edema (ALI/CPE). [file 13054_2014_659_MOESM4_ESM.docx]

**Additional file 6: Table S3. Predictors of “Other” vs “ALI or CPE”**

| **Predictor** | **Beta** | **SE** | **OR** | **95%-CI** | **P-Value** |
| --- | --- | --- | --- | --- | --- |
| Intercept | 0.19 | 0.27 | 1.21 | (0.72 to 2.0) | 0.48 |
| Age <45 years | -0.33 | 0.48 | 0.72 | (0.28 to 1.86) | 0.50 |
| History of Heart Failure | 0.40 | 0.36 | 1.49 | (0.74 to 3.0) | 0.26 |
| History of Coronary Artery disease | -0.43 | 0.35 | 0.65 | (0.33 to 1.29) | 0.22 |
| ST Changes | -0.51 | 0.41 | 0.60 | (0.27 to 1.34) | 0.21 |
| Sepsis or Pancreatitis | -0.11 | 0.31 | 0.90 | (0.49 to 1.63) | 0.73 |
| Pneumonia | 0.24 | 0.36 | 1.27 | (0.63 to 2.5) | 0.50 |
| Aspiration | 0.71 | 0.59 | 2.0 | (0.63 to 6.5) | 0.23 |
| Alcohol Abuse x ALI Risk Factor* | -0.44 | 0.64 | 0.64 | (0.18 to 2.3) | 0.49 |
| Chemotherapy | 0.17 | 0.39 | 1.19 | (0.55 to 2.6) | 0.66 |
| SpO_2_/FiO_2_-ratio <235 at 6 hours after onset of acute respiratory failure | -0.99 | 0.28 | 0.37 | (0.22 to 0.64) | <0.001 |

*ALI Risk Factor = Sepsis, Pancreatitis, Pneumonia, Aspiration

AUC = 0.66, 95%-CI=0.59 to 0.73
