# Supplementary material for: Decision support tool for differential diagnosis of Acute Respiratory Distress Syndrome (ARDS) vs Cardiogenic Pulmonary Edema (CPE): a prospective validation and meta-analysis
Source: Crit Care. 2014 Nov 29;18(6):659. doi: 10.1186/s13054-014-0659-x (PMC4277656; doi:10.1186/s13054-014-0659-x)
Supplement: Additional file 5 — Details about the Hosmer-Lemeshow test. [file 13054_2014_659_MOESM5_ESM.docx]

**Additional file 7. Detailed results from the Hosmer-Lemeshow test**

One way to calculate the probability of ALI vs CPE for a given score sum is to first regress the observed outcome Y (ALI vs CPE) against the prediction score (in the study cohort) using the following equation:

logit(Y) = b_0_ + b_1_*prediction score + e_i_

where e_i_ is the error term for subject i

Assuming the model fits the data well (“goodness-of-fit”), one can then calculate the probability of ALI vs CPE relatively easy for a given score sum as:

P(Y) = expit(b_0_ + b_1_*score sum) = exp(b_0_ + b_1_*score sum)/[1+exp(b_0_ + b_1_*score sum)]

To compute P(Y) when including the k-th polynomial of the prediction score in the logistic regression model, replace in all equations above the terms *b_1_*prediction score* and *b_1_*score sum* with


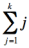
 [ b_j_*I(prediction score)_j_ ] where j = 1,…, k

To assess goodness-of-fit, the Hosmer-Lemeshow (HL) test stratifies the cohort by the deciles of predicted probability for the outcome, and then tests for significant differences between observed vs predicted number of cases with the outcome across strata using a Chi^2^-statistic. Thus a small P-Value indicates lack of fit, suggesting that a different function of the independent variable may be needed (e.g. additional quadratic and/or cubic terms). However, the HL-test has been criticized for several reasons:

- the stratification into deciles is arbitrary, using quintiles or other n-tiles can substantially change the results of the test
- more flexible functions (quadratic, cubic) allowing a “better” fit do not necessarily increase the P-Value as one would expect
- the P-Value is also a function of the sample size, thus in a large enough sample the test will always identify *statistically* significant differences (the question is as always if these differences are also *clinically* relevant)

For a more complete discussion see Allister, P. (2013), Why I don’t trust the Hosmer-Lemeshow test for logistic regression( <http://www.statisticalhorizons.com/hosmer-lemeshow> , last accessed on 04/16/2014).

Below are the detailed results for different logistic regression models using increasingly flexible functions of the prediction score, and their goodness-of-fit as assessed by the HL-test:

1.) Prediction Score modelled as a linear term (HL, P=0.01)

Standard Wald

Parameter DF Estimate Error Chi-Square Pr > ChiSq

Intercept 1 -0.7634 0.2293 11.0844 0.0009

FINAL_MODEL_SCORE 1 0.3723 0.0698 28.4514 <.0001

Partition for the Hosmer and Lemeshow Test

CPE0_ALI1 = 1 CPE0_ALI1 = 0

Group Total Observed Expected Observed Expected

1 14 2 2.08 12 11.92

2 15 1 3.16 14 11.84

3 9 2 2.40 7 6.60

4 17 2 5.40 15 11.60

5 21 13 8.43 8 12.57

6 15 12 8.12 3 6.88

7 14 10 9.06 4 4.94

8 16 11 11.91 5 4.09

9 14 9 12.06 5 1.94

10 10 10 9.38 0 0.62

Hosmer and Lemeshow Goodness-of-Fit Test

Chi-Square DF Pr > ChiSq

20.0777 8 0.0100

2.) Prediction Score modelled as a quadratic term (HL, P=0.047)

Standard Wald

Parameter DF Estimate Error Chi-Square Pr > ChiSq

Intercept 1 -0.7612 0.2413 9.9551 0.0016

FINAL_MODEL_SCORE 1 0.5346 0.1129 22.4242 <.0001

FINAL_MOD*FINAL_MODE 1 -0.0263 0.0120 4.8001 0.0285

Partition for the Hosmer and Lemeshow Test

CPE0_ALI1 = 1 CPE0_ALI1 = 0

Group Total Observed Expected Observed Expected

1 14 2 1.23 12 12.77

2 15 1 2.47 14 12.53

3 9 2 2.20 7 6.80

4 17 2 5.41 15 11.59

5 21 13 9.12 8 11.88

6 15 12 9.00 3 6.00

7 15 11 10.54 4 4.46

8 16 11 12.38 5 3.62

9 15 10 12.66 5 2.34

10 8 8 6.97 0 1.03

Hosmer and Lemeshow Goodness-of-Fit Test

Chi-Square DF Pr > ChiSq

15.6917 8 0.0470

3.) Prediction Score modelled as a cubic term (HL, P=0.04)

Standard Wald

Parameter DF Estimate Error Chi-Square Pr > ChiSq

Intercept 1 -0.5747 0.2831 4.1219 0.0423

FINAL_MODEL_SCORE 1 0.7581 0.1991 14.4950 0.0001

FINAL_MOD*FINAL_MODE 1 -0.1311 0.0720 3.3128 0.0687

FINAL_*FINAL_*FINAL_ 1 0.00840 0.00657 1.6363 0.2008

Partition for the Hosmer and Lemeshow Test

CPE0_ALI1 = 1 CPE0_ALI1 = 0

Group Total Observed Expected Observed Expected

1 14 2 0.42 12 13.58

2 15 1 1.73 14 13.27

3 9 2 2.19 7 6.81

4 17 2 6.12 15 10.88

5 21 13 10.75 8 10.25

6 15 12 9.77 3 5.23

7 14 10 9.82 4 4.18

8 16 11 11.64 5 4.36

9 14 9 10.81 5 3.19

10 10 10 8.75 0 1.25

Hosmer and Lemeshow Goodness-of-Fit Test

Chi-Square DF Pr > ChiSq

16.2418 8 0.0390
