# Supplementary material for: Decision support tool for differential diagnosis of Acute Respiratory Distress Syndrome (ARDS) vs Cardiogenic Pulmonary Edema (CPE): a prospective validation and meta-analysis
Source: Crit Care. 2014 Nov 29;18(6):659. doi: 10.1186/s13054-014-0659-x (PMC4277656; doi:10.1186/s13054-014-0659-x)
Supplement: Additional file 6 — Post hoc analysis of referral status. [file 13054_2014_659_MOESM6_ESM.docx]

**Additional file 8. Post-hoc analysis**

**Figure A. Retrospective validation cohort: Percent (95%-confidence interval) ALI cases observed (light) vs expected (dark) for each of the eight previously published prediction score categories.** The number/percent of expected ALI cases is based on the observed percentages of ALI cases across the eight score categories in the development cohort, which is used by the online calculator to translate a given patient’s score sum into the predicted probability of ALI vs CPE.[Schmickl et al, Chest 2011] Based on a Chi^2^-test with 7 degrees of freedom there were significant differences in observed vs expected number of ALI cases overall (P=0.004).


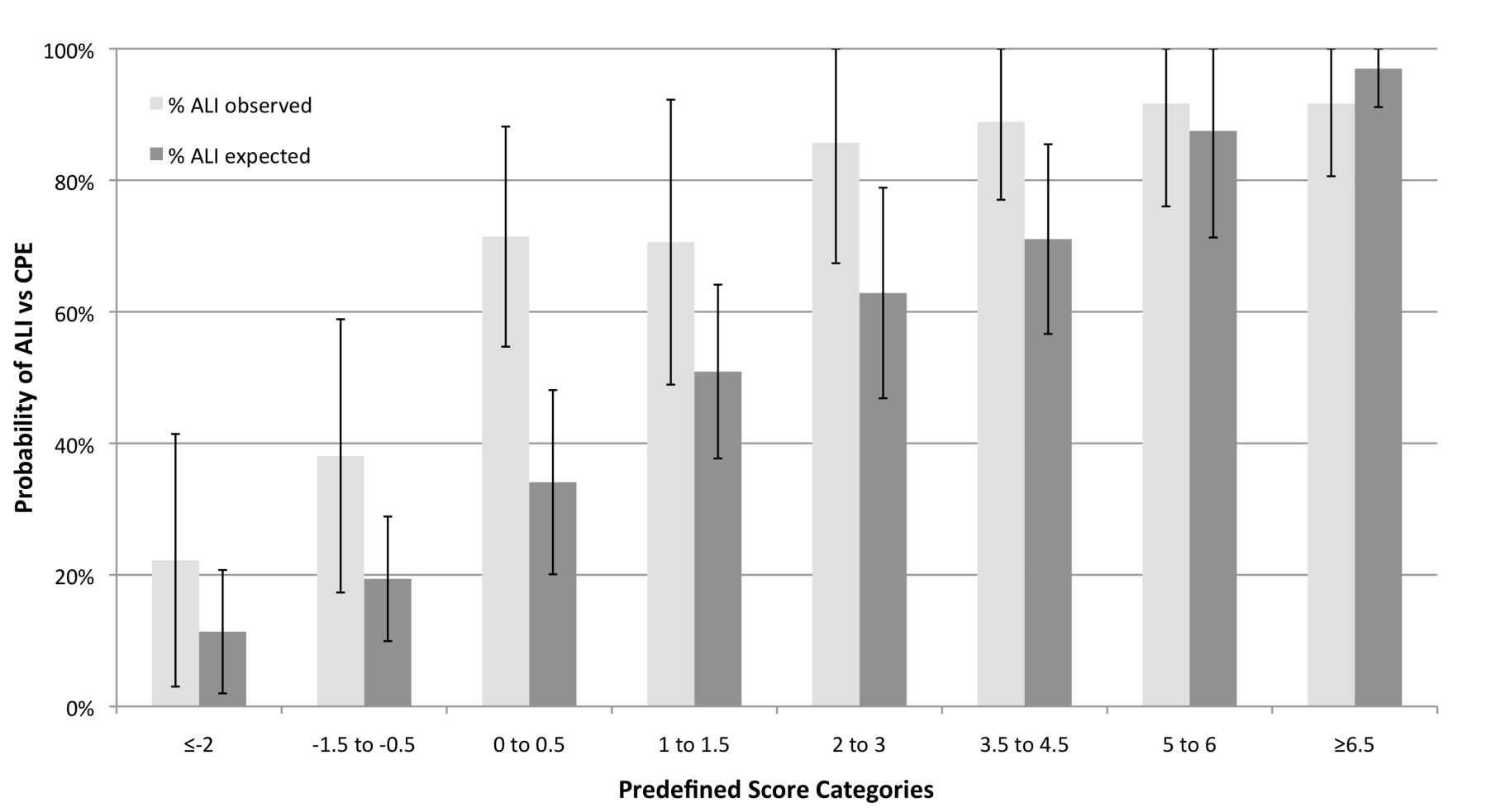


**Table A. Overview over prevalence and percentage of referral patients across the three cohorts**

| **Cohort** | **Prevalence ALI vs CPE** | **% referral patients** |
| --- | --- | --- |
| Development cohort | 47% | 0% |
| Retrospective validation cohort | 70% | 100% |
| Prospective validation cohort | 50% | 79% |

**Table B. Logistic regression model in the combined development and retrospective validation cohort: Referral status is a significant predictor of ALI vs CPE independently of the prediction score. The AUC improves slightly.**

| **Predictor** | **Beta** | **SE** | **OR** | **95%-CI** | **P-Value** |
| --- | --- | --- | --- | --- | --- |
| Intercept | -0.69 | 0.14 | 0.50 | (0.38 to 0.66) | <0.001 |
| Prediction Score | 0.47 | 0.05 | 1.60 | (1.46 to 1.76) | <0.001 |
| Referral patient | 1.00 | 0.24 | 2.71 | (1.69 to 4.35) | <0.001 |

**AUC=0.83 (0.79 to 0.86)**

**Table C. Logistic regression model in the prospective validation cohort. Referral status is not a significant predictor of ALI vs CPE independently of the prediction score. The AUC does not change.**

| **Predictor** | **Beta** | **SE** | **OR** | **95%-CI** | **P-Value** |
| --- | --- | --- | --- | --- | --- |
| Intercept | -1.35 | 0.47 | 0.26 | (0.10 to 0.65) | 0.004 |
| Prediction Score | 0.39 | 0.07 | 1.47 | (1.28 to 1.70) | <0.001 |
| Referral patient | 0.71 | 0.49 | 2.04 | (0.79 to 5.3) | 0.14 |

**AUC=0.80 (0.73 to 0.88)**
