# Supplementary material for: Decision support tool for differential diagnosis of Acute Respiratory Distress Syndrome (ARDS) vs Cardiogenic Pulmonary Edema (CPE): a prospective validation and meta-analysis
Source: Crit Care. 2014 Nov 29;18(6):659. doi: 10.1186/s13054-014-0659-x (PMC4277656; doi:10.1186/s13054-014-0659-x)
Supplement: Additional file 7: Table S4. — Sensitivity and specificity based on meta-analysis. [file 13054_2014_659_MOESM7_ESM.docx]

**Additional file 7: Table S4. Meta-analysis: the prediction score’s estimated sensitivity and specificity for ALI vs CPE at five different cut-offs based on combined data from the current and two previous cohorts (total n=638).** For CPE vs ALI the cut-off definitions, sensitivity and specificity are reversed (e.g. if all patients with a score ≤3 are classified as CPE, then the sensitivity and specificity for CPE vs ALI will be 89% and 50%, respectively)

|  |  | **Meta-Cohort**  **(DC + RVC + PVC)** | | | | |  |
| --- | --- | --- | --- | --- | --- | --- | --- |
|  |  |  | | | | |  |
| **Cut-off** |  | **Sensitivity**  **(95%-CI)** | |  | **Specificity**  **(95%-CI)** | |  |
| **> -1.5** |  | 94 | (92 to 97) |  | 37 | (32 to 43) |  |
| **> -0.5** |  | 90 | (87 to 93) |  | 52 | (46 to 57) |  |
| **> 0** |  | 81 | (76 to 85) |  | 68 | (62 to 73) |  |
| **> 1** |  | 66 | (61 to 71) |  | 81 | (76 to 85) |  |
| **> 2** |  | 57 | (52 to 63) |  | 84 | (80 to 88) |  |
| **> 3** |  | 50 | (45 to 55) |  | 89 | (85 to 92) |  |

Abbreviations: 95%-CI=95%-Confidence Interval, DC development cohort, RVC retrospective validation cohort, PVC prospective validation cohort
